# Exploring the phenotype of Italian patients with ALS with intermediate *ATXN2* polyQ repeats

**DOI:** 10.1136/jnnp-2022-329376

**Published:** 2022-08-25

**Authors:** Adriano Chio, Cristina Moglia, Antonio Canosa, Umberto Manera, Maurizio Grassano, Rosario Vasta, Francesca Palumbo, Salvatore Gallone, Maura Brunetti, Marco Barberis, Fabiola De Marchi, Clifton Dalgard, Ruth Chia, Gabriele Mora, Barbara Iazzolino, Laura Peotta, Bryan Traynor, Lucia Corrado, Sandra D'Alfonso, Letizia Mazzini, Andrea Calvo

**Affiliations:** 1 'Rita Levi Montalcini' Department of Neuroscience, University of Turin, Torino, Italy; 2 Neurology 1, Azienda Ospedaliero Universitaria Città della Salute e della Scienza di Torino, Torino, Italy; 3 Genetics, Azienda Ospedaliero Universitaria Città della Salute e della Scienza di Torino, Torino, Italy; 4 Neurology, Azienda Ospedaliero-Universitaria Maggiore della Carità, Novara, Italy; 5 Department of Anatomy, Physiology & Genetics, Uniformed Services University of the Health Sciences, Bethesda, Maryland, USA; 6 The American Genome Center, Collaborative Health Initiative Research Program, Uniformed Services University of the Health Sciences, Bethesda, Maryland, USA; 7 Neuromuscular Diseases Research Section, Laboratory of Neurogenetics, National Institute on Aging, NIH, Porter Neuroscience Research Center, Bethesda, Maryland, USA; 8 Neuromuscular Diseases Research Section, Laboratory of Neurogenetics, National Institute on Aging, Bethesda, Maryland, USA; 9 Department of Neurology, Johns Hopkins, Baltimore, Maryland, USA; 10 Department of Health Sciences Interdisciplinary Research Center of Autoimmune Diseases, University of Eastern Piedmont Amedeo Avogadro School of Medicine, Novara, Italy

**Keywords:** ALS, GENETICS

## Abstract

**Objective:**

To detect the clinical characteristics of patients with amyotrophic lateral sclerosis (ALS) carrying an intermediate *ATXN2* polyQ number of repeats in a large population-based series of Italian patients with ALS.

**Methods:**

The study population includes 1330 patients with ALS identified through the Piemonte and Valle d’Aosta Register for ALS, diagnosed between 2007 and 2019 and not carrying *C9orf72, SOD1, TARDBP* and *FUS* mutations. Controls were 1274 age, sex and geographically matched Italian subjects, identified through patients’ general practitioners.

**Results:**

We found 42 cases and 4 controls with≥31 polyQ repeats, corresponding to an estimated OR of 10.4 (95% CI 3.3 to 29.0). Patients with≥31 polyQ repeats (ATXN2+) compared with those without repeat expansion (ATXN2−) had more frequently a spinal onset (p=0.05), a shorter diagnostic delay (p=0.004), a faster rate of ALSFRS-R progression (p=0.004) and King’s progression (p=0.004), and comorbid frontotemporal dementia (7 (28.0%) vs 121 (13.4%), p=0.037). ATXN2+ patients had a 1-year shorter survival (ATXN2+ patients 1.82 years, 95% CI 1.08 to 2.51; ATXN2− 2.84 years, 95% CI 1.67 to 5.58, p=0.0001). *ATXN2* polyQ intermediate repeats was independently related to a worse outcome in Cox multivariable analysis (p=0.006).

**Conclusions:**

In our population-based cohort, ATXN2+ patients with ALS have a distinctive phenotype, characterised by a more rapid disease course and a shorter survival. In addition, ATXN2+ patients have a more severe impairment of cognitive functions. These findings have relevant implications on clinical practice, including the possibility of refining the individual prognostic prediction and improving the design of ALS clinical trials, in particular as regards as those targeted explicitly to *ATXN2*.

**WHAT IS ALREADY KNOWN ON THIS TOPIC**

- An intermediate-length CAG number of repeats (encoding≥31 glutamines, polyQ) in the *ataxin 2* (ATXN2) gene is recognised to be associated with an increased risk of developing amyotrophic lateral sclerosis (ALS). However, very few is known about the phenotypic characteristics of patients with *ATXN2* polyQ intermediate number of repeats.

**WHAT THIS STUDY ADDS**

- Patients with *ATXN2* polyQ intermediate number of repeats had more commonly a spinal onset and were characterised by a more rapid clinical course, as shown by a 1.5-fold ALSFRS-R progression and a significantly higher King’s progression at the time of diagnosis compared with patients without the expansion. In addition, patients with *ATXN2* PolyQ intermediate number of repeats were more frequently affected by frontotemporal impairment.

**WHAT THIS STUDY MAY AFFECT RESEARCH, PRACTICE, OR POLICY**

- The identification of the specific phenotypic characteristics of patients with ALS with *ATXN2* polyQ intermediate number of repeats has many implications, including the possibility of refining the individual prognostic prediction and improving the design of ALS clinical trials, including those targeted explicitly to *ATXN2*.

## Introduction

Amyotrophic lateral sclerosis (ALS) is a multisystem disorder of adult life characterised by progressive degeneration of upper and lower motor neurons and frontotemporal cortex neurons. Several genes have been related to this fatal neurodegenerative disorder, accounting for 10%–20% of ALS cases.^1 2^ Among these, an intermediate-length CAG number of repeats (encoding ≥31 glutamines, polyQ) in the *ataxin 2* (*ATXN2*) gene, already known as the cause of spinocerebellar ataxia type 2 (characterised by a number of polyQ≥38), is recognised to be associated with an increased risk of developing ALS and has been reported to be a modifier of survival.^3 4^ More recently, it has been reported that an intermediate number of *ATXN2* polyQ repeats can also be a modifier of frontotemporal dementia (FTD) phenotype.^5 6^ Nevertheless, the phenotypic characteristics of patients with ALS with intermediate-length CAG repeats in the *ATXN2* gene are still incompletely understood. This study aimed at detecting the clinical characteristics of patients with ALS carrying an intermediate number of *ATXN2* polyQ repeats in a large population-based series of Italian patients with ALS.

## Methods

The study population includes 1487 patients with ALS identified through the Piemonte and Valle d’Aosta Register for ALS, a prospective population-based register active since 1995. The characteristics of the register have been reported elsewhere.^7^ For the present paper, we considered ALS cases diagnosed between 2007 and 2019. Patients met the El Escorial revised diagnostic criteria for definite, probable and probable laboratory-supported ALS.^8^

ALSFRS-R mean monthly decline (∆ALSFRS-R) was calculated using the following formula: (*48 – ALSFRS-R score at diagnosis)/(months from onset to diagnosis*). Similarly, weight mean monthly decline (∆Weight) as (*Weight at diagnosis – healthy body weight)/(months from onset to diagnosis*). Finally, to have a proxy of disease spread, we calculated the mean/monthly decline of King’s staging as (*King’s staging at diagnosis)/(months from onset to diagnosis*).

A total of 928 patients underwent an extensive cognitive battery at the time of diagnosis. These cases were classified into five categories according to the Consensus Criteria for diagnosing frontotemporal cognitive and behavioural syndromes in ALS.^9^ The battery assessed executive function, memory, visuospatial function, language and social cognition using the following tests Letter Fluency test; Category Fluency Test; Frontal Assessment Battery; Trail Making Test A, B and B-A; Rey-Osterrieth Complex Figure Test, immediate (IR) and delayed recall (DR); Rey Auditory Verbal Learning Test (RAVL), immediate (IR) and DR; BSRT, immediate (IR) and DR; Digit Span Forward and Backward; Raven’s Colored Progressive Matrices (CPM47); Story-Based Empathy Task; and Mini-Mental State Examination. The raw scores of all tests were age, sex and education-corrected using the more recent Italian normative.^10^

Neurobehavioral dysfunction was determined with the Frontal Systems Behavior Scale (FrSBe), using the Family-form evaluated by a close relative/caregiver (scores: normal≤59, borderline 60–64; pathological≥65). For this study, we considered the change in points for each of the three domains of FrSBe (apathy, disinhibition, executive) from *before-disease* to *disease* scores. If a subject had scores reflecting a frontal systems abnormality both in the premorbid and post-illness forms, they were considered pathological only if there was an increase of≥10 points at the T-score between the two states.^10^ Anxiety and depression were assessed using the Hospital Anxiety and Depression Scale; the item ‘I feel slowed down’ was discussed with patients to have them not refer to physical disability.^11^

### Controls

Controls were 1274 age, sex and geographically matched Italian subjects, identified through patients’ general practitioners.

### Genetic analysis

All patients included in the study were tested for *SOD1* (all exons), *TARDBP* (exon 6), *FUS* (exons 14 and 15) mutations, and *C9ORF72* intronic expansion using the methods described elsewhere.^12^ However, since 1180 cases underwent whole-genome sequencing, no mutation in the other exons of *TARDBP* and *FUS* were found.

### ATXN2 CAG repeat analysis

In 434 cases and 509 controls, *ATXN2* CAG repeat in exon 1 (NM_002973.3) was amplified using a fluorescent primer and sized by capillary electrophoresis on an ABI3130 genetic analyzer (Applied Biosystems, Foster City, California, USA). In 1043 cases and 765 controls, *ATXN2* CAG repeats were detected through next-generation sequencing. All subjects were screened for the *C9orf72* intronic expansion using a standard repeat-primed PCR^13^ (Renton *et al*. 2011 PMID: 21944779). Repeat lengths of≥30 units with the characteristic sawtooth pattern were considered pathogenic. Whole-genome sequencing was performed at The American Genome Center located at the Uniformed Services University, Bethesda, Maryland, USA. Libraries were prepared using TruSeq DNA PCR-Free High Throughput Library Prep Kit (Illumina) per the manufacturer’s instructions. Sequencing was performed on an Illumina HiSeqX10 sequencer using paired-end 150 base pair reads. A significant advantage of next-generation sequencing is its ability to reliably assay repeat expansions, such as those in *C9orf72* and *ATXN2. ATXN2* CAG repeats were deemed intermediate if they were in the 31–38 range. ExpansionHunter—Targeted software (V.3.0.1) was used to estimate repeat lengths of known, disease-causing expansions in samples that had undergone whole-genome sequencing.^14^ This algorithm has been validated using experimentally confirmed samples carrying the *C9orf72* and *ATXN2* repeat expansions. In particular, cases with≥31 polyQ repeats were identified correctly with both PCR and ExpansionHunter.

### Statistical methods

Multivariable analysis for survival was performed with the Cox proportional hazards model (stepwise backward) with a retention criterion of p value<0.1. A p value<0.05 was considered significant. Statistical analyses were carried out using the SPSS V.26.0 statistical package (SPSS).

## Results

We assessed for intermediate *ATXN2* polyQ repeats 1487 patients with ALS diagnosed in Piemonte e Valle d’Aosta between 2007 and 2019. Of these, 157 were subsequently excluded from the present analysis because they carried a genetic mutation of one of the most common ALS-related genes (*C9orf72*, 97; *SOD1*, 32; *TARDBP,* 21; *FUS*, 6). We decided to exclude C9orf72 patients from this analysis because we found in a previous paper that in patients with this mutation intermediate *ATXN2* polyQ repeats do not modify survival.^15^ As for *SOD1, TARDBP* and *FUS*, we chose to also exclude these cases because of the strong influence of these genes on survival. The study was therefore performed on 1330 patients. Controls were 1274 subjects matched to cases by age, gender and geographical origin. Patients and controls did not differ for the main demographic variables (online supplemental table 1).

10.1136/jnnp-2022-329376.supp1 Supplementary data

In our cohort, we found 46 cases and 44 controls with 27 to 30 polyQ repeats, and 42 cases and 4 controls with≥31 polyQ repeats. The estimated OR for 27–30 polyQ was 0.99 (95% CI 0.66 to 1.52). The estimated OR for≥31 polyQ was 10.4 (95% CI 3.3 to 29.0). The 4 controls had 31 (3) and 32 (1) polyQ repeats. Their age ranged between 55 and 72 years. They were neurologically normal and they had no family history for neurodegenerative diseases or ataxia. The number of polyQ repeats in patients was 20 (31 repeats), 12 (32 repeats), 4 (33 repeats), 3 (34 repeats), 2 (35 repeats) and 1 (38 repeats).

*Phenotype of ATXN2+ patients* (table 1). Patients with intermediate *ATXN2* polyQ repeats≥31 polyQ repeats (ATXN2+) and those without expansion (<31 polyQ repeats) (ATXN2−) did not differ for the age at onset but ATXN2+ had more frequent spinal onset (p=0.05). In addition, ATXN+ patients had a shorter diagnostic delay (p=0.004) and a faster rate of progression as measured by ΔALSFRS-R (p=0.004) and ΔKing’s (p=0.004).

**Table 1:** Demographic and clinical characteristics of patients according to *ATXN2* PolyQ intermediate number of repeats (ATXN2+, PolyQ≥31; ATXN2−, PolyQ≤30)

	ATXN2+ (n=42)	ATXN2− (n=1288)	P value
Age at onset (median, IQR)	69.6 (63.5–75.7)	68.3 (60.3–74.4)	0.15
Gender (female)	15 (35.7%)	575 (44.6%)	0.25
Site of onset (bulbar)	7 (16.7%)	393 (30.5%)	0.05
Diagnostic delay (months, IQR)	6.0 (3.94–10.03)	9.04 (5.88–13.97)	0.004
ALSFRS-R score at diagnosis (median, IQR)	42 (34.75–44)	42 (37–45)	0.13
ΔALSFRS-R (median points/month, IQR)	1.00 (0.50–1.99)	0.66 (0.31–1.33)	0.004
FVC% at diagnosis (median, IQR)*	89 (74–105)	91 (72–104)	0.81
ΔWeight (kg/month, median, IQR)†	0.50 (0–1.26)	0.28 (0–0.96)	0.87
MiToS stage at diagnosis (0/1/2/3/4)	27/12/2/1/0	858/374/42/10/2	0.80
King’s state at diagnosis (1/2/3/4)	14/14/11/3	530/409/293/50	0.59
ΔKing’s (median points/month, IQR)	0.25 (0.17–0.53)	0.19 (0.10–0.34)	0.004
ALS-FTD‡	7 (28.0%)	121 (13.4%)	0.037

*FVC, 1222 (ATXN2+, 38; ATXN2−, 1184).

†Weight 1288 (ATXN2+, 40; ATXN2−, 1246).

‡928 cases (ATXN2+, 25, ATXN2−, 903)

A total of 928 patients (25 ATXN2+ and 903 ATXN2−) and 129 controls underwent cognitive examination. The demographic and clinical characteristics of these patients did not differ from that of the overall cohort (online supplemental table 2). ATXN2+ patients were more commonly diagnosed as ALS-FTD (7 (28.0%) vs 121 (13.4%), p=0.037) (table 2).

**Table 2:** Frequency of cognitive impairment classified according to the consensus criteria for the diagnosis of frontotemporal cognitive and behavioural syndromes in ALS^9^

	ATXN2+ (n=25)	ATXN2- (n=903)
Cognitively normal ALS	10 (40%)	483 (53.5%)
ALSbi	–	72 (8%)
ALSci	7 (28%)	164 (18.2%)
ALScbi	1 (4%)	63 (7%)
ALS-FTD	7 (28%)	121 (13.4%)

In patients with ALS with *ATXN2* PolyQ intermediate number of repeats ≥31 (ATXN2+) compared with patients with PolyQ ≤30 (ATXN2-). ALS-FTD was significantly more frequent in ATXN2+ patients (p=0.037).

ALS, amyotrophic lateral sclerosis; ALSbi, ALS with behavioural impairment; ALScbi, ALS with cognitive and behavioural impairment; ALSci, ALS with cognitive impairment; ALS-FTD, ALS with comorbid FTD.

When assessing the differences in specific tests, excluding the 128 patients with ALS-FTD, we found a significantly worse performance of ATXN2+ patients in the RAVL − Delayed Recall (p=0.023), the BSRT − Immediate Recall (p=0.044), and in the Executive Functions domain of ECAS (p=0.034) (online supplemental tables 3 and 4). No differences were found in the behavioural function assessed with FrSBe. Finally, no differences were found in anxiety, while depression was significantly more severe in ATXN2+ patients (p=0.04).

### Survival analysis

**Figure 1:**
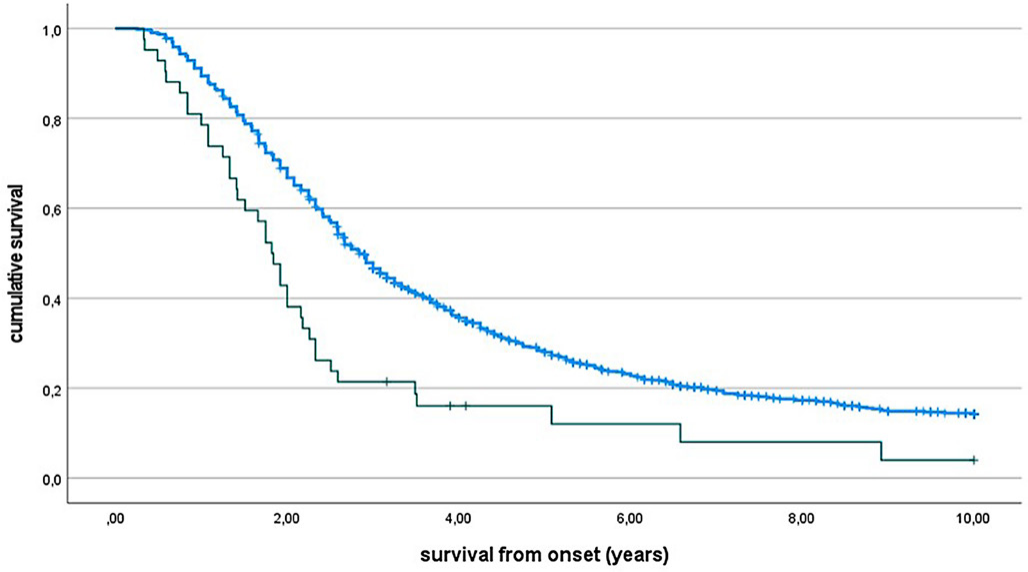
Survival from onset according to *ATXN2* polyQ intermediate number of repeats. PolyQ≥31 (green line) versus PolyQ≤30 (blue line). Ticks indicate censored patients. P<0.0001.

ATXN2+ patients had a 1 year shorter survival (median survival time for ATXN2+ patients = 1.82 years, 95% CI 1.08 to 2.51; ATXN2− =2.84 years, 95% CI 1.67 to 5.58, p=0.0001, figure 1). The Cox multivariable analysis confirmed that the ATXN2 polyQ intermediate number of repeats was independently related to a worse outcome compared with non-expanded patients in our cohort (p=0.006) (table 3). A separate analysis including only the patients not assessed in our previous paper^3^ gave similar results (data not shown).

**Table 3:** Patients’ survival

Variable	Value	HR (95% CI)	P value
Age at onset (years)	Per each year of age at onset	1.030 (1.024 to 1.037)	0.0001
Diagnostic delay (months)	Per each month of delay	0.953 (0.945 to 0.962)	0.0001
Site of onset	Spinal Bulbar	1 (reference) 1.45 (1.27–1.67)	0.0001
ΔALSFRS-R	Per each point loss/month	1.25 (1.20 to 1.29)	0.0001
ΔKing’s	Per each point loss/month	1.56 (1.16 to 2.09)	0.003
*ATXN2* polyQ	<31 ≥31	1 (reference) 1.58 (1.14 to 2.19)	0.006

Cox multivariable analysis.

### Analysis of oligogenicity

All but two patients with≥31 polyQ repeats underwent whole-genome sequencing. We extracted variant information for 47 genes previously implicated in ALS pathogenesis (see online supplemental table 5 for the list of extracted genes). The following six mutations of ALS-related genes, all in heterozygosis, of ALS-related genes were detected (one for each patient): *OPTN*: p.L111R; *SETX*: p.H476R; *CCNF*: p.R123H; *EWSR1*: p.A132S; *SETX*: p.V919I; *DTCN1*: p.A354V. Allele frequencies and predicted functional effects of identified genetic variants are reported in online supplemental table 6. Patients carrying one of these mutations (n=6) did not differ from those not carrying other genetic mutations (n=34) for any clinical characteristic but the median weight decline (Δweight) (see online supplemental table 7).

## Discussion

We have assessed a large population-based cohort of Italian patients with ALS without mutations for *C9orf72, SOD1, TARDBP* and *FUS* genes to identify the clinical signature of *ATXN2* polyQ intermediate number of repeats. We have found that ATXN2+ patients, despite having more commonly spinal onset, were characterised by a more rapid clinical course, as shown by (1) a 1.5-fold ΔALSFRS-R at the time of diagnosis compared with ATXN2- patients; (2) a shorter diagnostic delay, a factor related to a faster disease progression; and (3) a significantly higher ΔKing’s, indicating a more rapid spreading of symptoms from one to three body regions. The greater aggressiveness of ALS in subjects with *ATXN2* polyQ intermediate number of repeats is reflected in the 1 year shorter survival (median survival time, 1.82 vs 2.84), confirming our previous findings.^3^ This result was independent of relevant prognostic factors in Cox multivariable analysis. Finally, patients with *ATXN2* PolyQ intermediate number of repeats were more frequently affected by frontotemporal impairment.

*ATXN2* polyQ intermediate number of repeats were first recognised as a risk factor for ALS in 2010 in a cohort of US patients (using a cut-off≥27)^16^ and subsequently confirmed in populations of different ethnic origin,^3^ with the only exception of South Africans.^17^ Although a length of 27–33 polyQ was initially considered significantly associated with ALS, later studies have shown that the cut-off is≥31 polyQ repeats. In our population, we found that the best risk cut-off for ALS is≥31 polyQ since the distribution of alleles in the 27–30 polyQ repeats range was substantially similar among patients and controls.

*ATXN2* is an RNA binding protein with an essential function in the nucleocytoplasmic shuttling of RNA and regulation of transcription.^18^ However, the pathogenic mechanism of *ATXN2* in ALS is unknown. It has been reported that *ATXN2* induces an increase of phosphorylated TDP-43 in the spinal anterior horn but not in motor cortex neurons of patients with ALS.^19^ Interestingly, our data show that the spinal phenotype is more common in patients with *ATXN2* polyQ intermediate number of repeats than those without expansion.

A recent paper has reported that ATXN+patients (cut-off limits≥31) did not show any survival difference compared with ATXN-.^20^ The discrepancy with the present study is likely related to the different nature of the two cohorts, that is, the prevalent, referral centers, population in the US study (as indicated by the young age at onset (~60 years), the median survival (~3.5 years) and finally the very low percentage of patients with ALS-FTD (3.8%)) and the incident population in our study. It is therefore possible that at least a part of fast progressor patients, including those who are ATXN+, have not been caught in the US study. Several papers have demonstrated that prevalent and incident populations strongly differ from the clinical point of view, including survival, supporting the notion that studies derived from clinical cohorts should be cautiously interpreted.^21 22^

A novel finding in this paper is the identification of a correlation between *ATXN2* polyQ intermediate number of repeats in ALS and cognitive impairment. ALS-FTD was two times more frequent among the ATXN2+ patients (28% vs 13.4%). Furthermore, some cognitive tests related to frontal function (RAVL – delayed recall, BSRT – immediate recall, and Executive Functions domain of ECAS) were significantly more compromised in ATXN2+ patients. Interestingly, *ATXN2* polyQ intermediate number of repeats (using a cut-off of≥27) have been recently proposed to be a modifier of the behavioural variant FTD phenotype, with earlier age at onset and more frequent parkinsonian and psychotic symptoms.^5 6 23^ However, no increased risk of developing behavioural variant FTD was reported by other studies.^24 25^ An antagonistic pleiotropic role in cognition of *ATXN2* has been identified, with a positive influence on verbal–numerical reasoning, reaction time, educational attainment and cognitive resilience,^26 27^ while in spinocerebellar ataxia 2 polyQ expansions are related to cognitive impairment in executive functions, memory and visuoconstructive skills.^28^ Finally, a postmortem study in patients with non-fluent primary progressive aphasia with 39 polyQ repeat expansion showed neuronal loss and gliosis, associated with a superficial laminar spongiosis, were severe in the superficial layers of the middle frontal gyrus, motor cortex, supramarginal gyrus, CA1 and the subiculum, but not in the cerebellum.^29^

One limitation of this study is that not all patients were tested for cognitive function. However, the clinical characteristics of tested and non-tested patients were similar, excluding a selection bias. A remarkable feature of our study is its population-based nature, including some 80% of incident patients.

In conclusion, in our population-based cohort of patients of Italian ancestry, we found that patients with ALS carrying an intermediate *ATXN2* polyQ number of repeats≥31 have a distinctive phenotype, characterised by a more rapid disease course, with a 1.5-fold increase of ΔALSFRS-R rate and a higher ΔKing’s. Compared with ATXN2− patients, this greater aggressiveness resulted in a 1 year reduction in survival. In addition, ATXN2+ patients have a more severe impairment of cognitive functions, with relative preservation of the behavioural domain. Identifying the specific phenotypic characteristics of patients with ALS with *ATXN2* polyQ intermediate number of repeats has many implications. These include the possibility of refining the individual prognostic prediction and improving the design of ALS clinical trials, including those targeted explicitly to *ATXN2*.

## Data Availability

Data are available upon reasonable request. Data will be available upon reasonable request by interested researchers.
