# Supplementary material for: Exploring the phenotype of Italian patients with ALS with intermediate ATXN2 polyQ repeats
Source: J Neurol Neurosurg Psychiatry. 2022 Aug 25;93(11):1216–20. doi: 10.1136/jnnp-2022-329376 (PMC9606535; doi:10.1136/jnnp-2022-329376)
Supplement: Supplementary data [file jnnp-2022-329376supp001.pdf]

Supplementary Table 1. Comparison of demographic and genotype data of ALS cases and controls

	**ALS cases**<br>**n=1330**	**Controls**<br>**n=1274**	**P value**
Median age (IQR), years	68.4 (60.6-74.4)	66.9 (59.0-71.8)	0.15
Sex (female, %)	590 (44.3%)	590 (46.3%)	0.32
Median education (IQR), years	8.0 (5.0-12.0)	8.2 (4.9-12.2)	0.32
ATXN2 polyQ ≥31	42 (3.3%)	4 (0.3%)	0.0001

Supplementary Table 2. Comparison of demographic and genotype data of ALS cases according to the study cohorts

	ALS cases – whole cohort (n=1330)	ALS cases- cognitive cohort (n=928)	p
Age at onset (median, IQR)	68.3 (60.4-74.4)	68.1 (60.3-73.9)	0.85
Gender (female)	590 (44.4%)	403 (43.4%)	0.66
Site of onset (bulbar)	400 (30.1%)	292 (31.5%)	0.48
Diagnostic delay (months, IQR)	9.04 (5.10-13.97)	9.04 (5.11-13.97)	0.94
ALSFRS-R score at diagnosis (median, IQR)	42 (37-45)	42 (38-45)	0.43
FVC% at diagnosis (median, IQW)	91 (72-104)	93 (74-106)	0.21
ΔALSFRS-R (median points/month, IQR)	0.67 (0.33-1.34)	0.60 (0.30-1.18)	0.12
ΔWeight (Kg/month, median, IQR)	0.28 (0-0.98)	0.25 (0-0.87)	0.37
MiToS stage at diagnosis (0/1/2/3/4)	887/386/44/11/2	650/252/20/5/1	0.30
King's state at diagnosis (1/2/3/4)	550/423/304/53	408/304/192/24	0.15
ΔKing's (median points/month, IQR)	0.20 (0.10-0.34)	0.19 (0.10-0.33)	0.28
ATXN2 PolyQ (n, %)	42 (3.2%)	25 (2.7%)	0.61

Supplementary Table 3. Median values (interquartile range) of age-, and education-corrected scores of cognitive tests in ALS patient according to ATXN2 status. 128 patients with FTD (7 ATXN2+ and 121 ATXN2-) have been excluded). P values are calculated with the Mann-Whitney U test.

**Test**	**ATXN-**<br>**(n=903)**	**ATXN+**<br>**(n=25)**	**p**
**MMSE**	27.5 (26.2-29.5)<br>n=903	28.2 (26.9-30)<br>n=25	0.30
**FAS**	28.8 (22.4-35.9)<br>n=836	28.7 (22.4-40.8)<br>n=22	0.68
**CAT**	19.3 (15.5-22.0)<br>n=836	18.8 (16.0-25.8)<br>n=22	0.51
**FAB**	14.9 (13.2-16.5)<br>n=778	15.2 (13.5-17.8)<br>n=22	0.50
**Digit Span FW**	5.7 (5.0-6.3)<br>n=789	5.6 (4.9-6.4)<br>n=19	0.79
**Digit Span BW**	3.9 (3.4-4.5)<br>n=789	4.3 (2.6-4.7)<br>n=19	0.78
**TMT A**	39 (25-61)<br>n=781	40 (26-76)<br>n=18	0.73
**TMT B**	81 (39-174)<br>n=781	74 (43-307)<br>n=18	0.74
**TMT B-A**	44 (12-115)<br>n=781	52 (12-1735)<br>n=18	0.73
**RAVL-IR**	39.2 (32.9-45.5)<br>n=569	37.0 (34.1 -39.1)<br>n=14	0.18
**RAVL-DR**	6.0 (4.0-9.0)<br>n=569	3.0 (2.0-6.0)<br>n=14	**0.023**
**BSRT-IR**	5.6 (4.5-6.8)<br>n=543	4.6 (3.5-5.4)<br>n=14	**0.044**
**BRRT-DR**	6.5 (5.0-7.5)<br>n=543	5.4 (4.3-7.2)<br>n=14	0.07
**ROCF-IR**	31.5 (26.3-34.3)<br>n=676	31.9 (27.4-34.3)<br>N=15	0.95

**ROCF-DR**	11.7 (7.7-16.4)<br>n=676	12.2 (6.9-16.8)<br>n=15	0.31
**CPM47**	28.5 (24.2-31.5)<br>n=823	30.0 (26.5-33.0)<br>n=15	0.20
**Clock**	4 (3-5)<br>n=755	4 (3-5)<br>n=22	0.78
**SET-IA**	4.2 (3.2-5.3)<br>n=150	3.2 (1.5-5.0)<br>n=6	0.30
**SET-CI**	4.3 (3.3-5.2)<br>n=150	4.7 (2.6-6.0)<br>n=6	0.75
**SET-EA**	4.2 (3.2-5.9)<br>n=150	3.3 (2.5-4.0)<br>n=6	0.18
**SET-GS**	13.1 (9.3-15.3)<br>n=150	10.4 (6.6-14.8)<br>n=6	0.38
**HADS-A**	7 (5-10)<br>n=795	10 (2-14)<br>n=16	0.43
**HADS-D**	5 (3-8)<br>n=795	9 (3.8-11)<br>n=16	**0.04**

* The scores of the Clock test are not corrected by age and education because no Italian normative data are available

MMSE, Mini-Mental State Examination; FAS, Letter Fluency test; CAT, Category Fluency Test; FAB, Frontal Assessment Battery; Digit Span FW, Digit Span Forward; Digit Span BW, Digit Span Backward; TMT A, Trail Making Test A; TMT B, Trail Making Test B; TMT B-A, Trail Making Test B-A; RAVL-IR, Rey Auditory Verbal Learning Test, Immediate Recall; RAVL-DR, Rey Auditory Verbal Learning Test, Delayed Recall; BSRT-IR, Babcock Story Recall Test, Immediate Recall; BSRT-DR, Babcock Story Recall Test, Delayed Recall ; ROCF-IR, Babcock Story Recall Test, Immediate Recall ; ROCF-DR, Babcock Story Recall Test, Delayed Recall ; CPM47, Raven's Colored Progressive Matrices; Clock, Clock Drawing Test; SET-IA, Story-Based Empathy Task – Intention Attribution; SET-CI, Story-Based Empathy Task – Causal Inference; SET-EA, Story-Based Empathy Task – Emotion Attribution; SET-GS, Story-Based Empathy Task – Global Score; HADS-A, Hospital Anxiety and Depression Scale - Anxiety; HADS-D, Hospital Anxiety and Depression Scale - Depression

Supplementary Table 4. Median values (interquartile range) of age-, and education-corrected scores of ECAS in ALS patient according to ATXN2 status. Patients with FTD have been excluded. P values are calculated with the Mann-Whitney U test.

**Domains**	**ATXN2- (n=291)**	**ATXN2+ (n=6)**	**p**
**Language**	25 (22-27)	25.5 (24.2-27.5)	0.44
**Fluency**	16 (12-18)	15 (14-17.5)	0.92
**Executive**	30 (22-38)	20.5 (20-24)	**0.034**
**Memory**	17 (14-19)	14.5 (13.2-16.5)	0.061
**Visuospatial**	12 (11-12)	11.5 (9.5-12)	0.67
**ALS specific functions**	70.5 (57-79)	62 (60-66)	0.11
**ALS non-specific functions**	28 (25-31)	26 (23.5-27.5)	0.25
**ECAS total score**	97 (82-110)	87.5 (84.8-93.3)	0.19

Supplementary Table 5. List of extracted genes

GENES			
ALS2	DCTN1	NEK1	SS18LL1
ANG	DNAJC7	OPTN	SPTLC1
ANXA11	ERBB4	PFN1	SQSTM1
ATXN2	ESWR1	PON1	TAF15
C21orf2	FIG4	PON2	TARDBP
C9orf72	FUS	PRPH	TBK1
CAMTA1	HNRNPA1	PGRN	TUBA4A
CCNF	HNRNPA2B1	SETX	UBQLN2
CEP112	KIF5A	SIGMAR1	UNC13A
CHCHD10	MAPT	SOD1	VAPB
CHMP2B	MATR3	SPAST	VCP
DAO	NEFH	SPG11	

Supplementary Table 6. Allele frequencies and predicted functional effects of identified genetic variants

Gene	ExonicFunc	AAChange.refGene	dbSNP	ExAC freq	gnomAD exome NFE	SIFT	Polyphen2 HVAR	LRT	MutationTaster	FATHMM	MetaSVM	MetaLR	CADD
CCNF	nonsynonymous SNV	exon5:c.G368A:p.R123H	rs371699142	4.24E-05	4.52E-05	T	P	D	D	T	T	T	25.1
DCTN1	nonsynonymous SNV	exon11:c.C1061T:p.A354V	.	.	.	D	P	D	D	T	D	D	28.4
EWSR1	nonsynonymous SNV	exon5:c.G394T:p.A132S	.	.	.	T	B	U	D	T	T	T	24.2
OPTN	nonsynonymous SNV	exon3:c.T332G:p.L111R	.	.	.	D	B	N	D	D	D	D	26.4
SETX	nonsynonymous SNV	exon10:c.A1427G:p.H476R	rs779742691	8.53E-06	9.34E-06	D	D	D	D	D	D	D	24.8
SETX	nonsynonymous SNV	exon10:c.G2755A:p.V919I	.	.	0	D	B	N	N	D	T	T	10.35

SIFT: D, Deleterious; T, tolerated: PolyPhen-2: D, Damaging; P, Possibly Damaging; B, Benign; LRT: D, Deleterious; N, neutral; U, Unknown; Mutation Taster, D, Deleterious; N, Neutral; FATHMM: D, Deleterious; T, Tolerated: MetaSVM: D, Deleterious; T, Tolerated; MetaLR: D, Deleterious; T, Tolerated

Supplementary Table 7. Demographic and clinical characteristics of patients with ≥31 *ATXN2* PolyQ intermediate number of repeats according to the presence of mutations in other genes

	**With other gene mutations (n=6)**	**Without other gene mutations (n=34)**	**p**
Age at onset (median, IQR)	71.5 (65.5-77.9)	68.3 (62.9-75.8)	0.52
Gender (female)	2 (33.3%)	12 (35.3%)	0.65
Site of onset (bulbar)	1 (14.7%)	5 (16.7%)	0.65
Diagnostic delay (months, IQR)	5.7 (2.5-10.5)	6.0 (3.9-9.9)	0.62
ALSFRS-R score at diagnosis (median, IQR)	40 (30-45)	42 (35-44)	0.96
ΔALSFRS-R (median points/month, IQR)	1.37 (0.55-4.41)	1.00 (0.49-1.85)	0.45
FVC% at diagnosis (median, IQR) *	77 (51-98)	89 (79-106)	0.19
ΔWeight (Kg/month, median, IQR) §	1.94 (0.4-3.10)	0.29 (0-1.03)	0.026
MiToS stage at diagnosis (0/1/2/3/4)	5/0/1/0	21/11/1/1	0.22
King's state at diagnosis (1/2/3/4)	2/1/2/1	12/12/8/2	0.67
ΔKing's (median points/month, IQR)	0.49 (0.19-1.10)	0.25 (0.17-0.51)	0.31
ALS-FTD ^	1 (33.3%)	5 (23.8%)	0.037

Median survival (years, IQR)	1.08 (0.84-2.26)	1.82 (1.25-2.59)	0.30

* available for 36 patients (5 with other mutations and 31 without other mutations)

§ available for 38 patients (6 with other mutations and 32 without other mutations)

^ available for 24 patients (3 with other mutations and 21 without other mutations)
